# When “ultrarapid” word-related motor activity is not faster than “early”

**DOI:** 10.3389/fnhum.2014.00842

**Published:** 2014-10-16

**Authors:** Liuba Papeo, Alfonso Caramazza

**Affiliations:** ^1^Center for Mind/Brain Sciences (CIMeC), University of TrentoRovereto, Italy; ^2^Department of Psychology, Harvard UniversityCambridge, MA, USA

**Keywords:** word semantics, motor processes in the brain, electrophysiology, grammatical class, conceptual representation

How do we understand words? On one current hypothesis, meaning is assigned through the retrieval of the sensory and/or motor information that contributes to define the word's referent, as we experience it in the environment. In the case of words denoting motor actions, the representation for action production contained in the motor system would be *directly* accessed from the visual/acoustic representation of a word (Pulvermüller, 2005). This direct perception-to-motor-mapping would be the key neural mechanism for understanding.

One line of thinking to address this hypothesis is the following: if motor activity occurs immediately (~200 ms) after word onset, it must be a correlate of direct access to semantically-relevant representations, as opposed to post-comprehension processing including epiphenomenal imagery.

Applying this reasoning, Shtyrov et al. (2014) used magnetoencephalography to measure the mismatch negativity (MMN) response to spoken Russian verbs and nouns denoting hand-, leg/foot-, and face-related actions, embedded as deviant trials in a stream of frequent pseudowords (standard trials). The MMN elicited by pseudorandom oddball sequences is considered a correlate of word-memory trace activation. The results showed increased response for hand-words in the left precentral region around the “compatible” motor site (hand-representation), followed by activation suppression in “incompatible” motor sites (leg/foot- and face-representation). The same pattern was obtained for leg/foot- and face-words (but see Ibáñez et al., 2013).

The relevance of these findings for the interpretation of word-related motor activity is in the timing of the effect: 85–125 ms from a word *disambiguation-point*. In the context of word processing, a semantic effect as early as 85 ms is certainly “ultrarapid,” as the authors remark, but it is also quite surprising, since similar latencies are typically associated with the sensory (acoustic) analysis of the stimulus (Friederici, 2002). The key to solving this apparent discrepancy is in the definition of “disambiguation-point,” which the authors took to be the onset of the suffix that distinguished the three word-categories Verb, Noun, and Pseudoword, otherwise identical. In other terms, the same root-morpheme (e.g., [brΛ's]) was used across the three conditions, followed by the suffix [-aj] for verbs ([brΛ'saj], *throwing*), [-ok] for nouns ([brΛ'sok], *a throw*) and [ɨm] for pseudowords ([brΛ'sɨm]). Using this criterion, the disambiguation-point occurred 261 ms after the word onset—the onset of the information (i.e., the suffix) that disambiguated the grammatical class of the stimulus. The latency of the word-related motor activation—85–125 ms—was measured from this point. But, is this information relevant to the theoretical issue under consideration here?

Recasting the motor effect reported by Shtyrov et al. in terms of a *standard* description that measures effect latencies from word onset, it would be in the range 346–386 ms—values more compatible with the classical view that motor activation is subsequent to semantic activation in non-motor areas of the brain. It is crucial, therefore, to determine which latency measure of the reported motor effect is appropriate for assessing the claim of ultrarapid semantic activation of the motor system: should it be from word onset or from the grammatical class disambiguation-point?

Since the purpose of the study was to determine the earliest point of semantic differentiation in neural responses, the relevant measure would be one that considers the point at which semantic information becomes available. In the context of the present study this is indeterminate, but it is certainly *before* the grammatical class disambiguation-point used by Shtyrov et al. The grammatical disambiguation-point distinguishes between two words with the same root-morpheme (e.g., [brΛ's], *throw*-). However, it is the root-morpheme that carries the core aspects of the words' semantics (*throw*, in this example) and, therefore, the grammatical class disambiguation implemented in this study does not provide information relevant to the point at which core semantic information becomes available. And, indeed, no difference between verbs and nouns was found in the MMN recorded from precentral motor cortex, confirming that the effect is driven by the meaning of the root-morpheme and not the grammatical class. In the light of these considerations, the relevant time window for interpretation of the motor effect should be considered as starting as soon as critical information about the root-morpheme becomes available, that is, from the word onset (Marslen-Wilson, 1987). In this way, the proper description of the timing of the semantic motor effect is as occurring in the range 346–386 ms after word onset, consistent with a post-comprehension account of the effect.

There are two other considerations to keep in mind in interpreting results such as those reported by Shtyrov et al. First, it has been shown that even early “semantic” precentral motor activity is mediated by neural activity in posterior temporal cortex. In a recent transcranial magnetic stimulation (TMS) study, disruption of visual word processing in a left temporal site, independently shown to be involved in semantic processing, abolished word-related precentral motor activity found as early as 250 ms after word onset (Papeo et al., 2014). This study highlights how evidence of early motor response to words does not mean evidence of direct (unmediated) access. Second, the claim of direct access to semantic motor representations remains unspecified both in terms of the content of such putative semantic representations and in terms of the mechanism of “direct” access (Mahon and Caramazza, 2008). Thus, for example, what is motor about the representation of “throw” in such uses as: throw down, throw a ball, throw a punch, throw a fit, throw up, throw around, throw a party, throw a fight, and on and on? And, what is direct about access of such representations when the input is something like “throw a party?”

## Conflict of interest statement

The authors declare that the research was conducted in the absence of any commercial or financial relationships that could be construed as a potential conflict of interest.
